# Total glucosides of paeony in cirrhosis: mechanisms of action via the gut–liver axis – a review

**DOI:** 10.3389/fgstr.2026.1981682

**Published:** 2026-10-27

**Authors:** Shulan Chen, Qiang Zheng

**Affiliations:** Department of Traditional Chinese Medicine, Zhangzhou Affiliated Hospital of Fujian Medical University, Zhangzhou, Fujian, China

**Keywords:** cirrhosis, farnesoid X receptor, gut microbiota, gut–liver axis, NLRP3 inflammasome, total glucosides of paeony

## Abstract

**Background & aim:**

Liver cirrhosis, the end-stage of chronic liver diseases, imposes a high global burden. Gut–liver axis dysregulation—microbiota imbalance, barrier failure, endotoxin translocation, bile acid disturbance, suppressed FXR signaling, and NLRP3 activation—drives cirrhosis progression. Total glucosides of paeony (TGP), a Paeonia lactiflora extract rich in paeoniflorin (>90%), possesses anti-inflammatory and immunomodulatory properties. However, a systematic review of TGP’s mechanisms via the gut–liver axis is lacking. This review aims to systematically summarize and critically evaluate the molecular mechanisms by which TGP modulates the gut–liver axis to treat cirrhosis, covering its effects on microbiota restoration, barrier repair, bile acid normalization, FXR reactivation, and dual inhibition of NF-κB and NLRP3 inflammatory pathways, thereby providing a comprehensive mechanistic framework for its therapeutic application.

**Main findings:**

Preclinical evidence shows TGP is associated with improved microbiota balance (increased Lactobacillus, Akkermansia, Lachnospira), Through short-chain fatty acids, the expression of tight junction proteins (ZO-1, Occludin, Claudin-3) may be upregulated, and the levels of endotoxin and bacterial translocation in the portal vein may be reduced. TGP normalizes primary-to-secondary bile acid conversion (CA/CDCA to DCA/LCA), thereby activating intestinal and hepatic farnesoid X receptor (FXR). Its main component, paeoniflorin, enhances FXR through PKCα/MAPK inhibition and SIRT1 upregulation. Activated FXR dually suppresses NF-κB (directly and via Nrf2/HO-1) and inhibits NLRP3 Ser295 phosphorylation, while reducing bile acid synthesis, achieving coordinated anti-inflammatory and barrier-protective effects.

**Conclusion:**

TGP may intervene in the vicious cycle of liver cirrhosis through a hierarchical cascading reaction - barrier repair, bile acid restoration, FXR activation, and dual NF-κB/NLRP3 inhibition. Despite predominantly preclinical data, TGP’s multi-target profile offers promising therapeutic potential; future large-scale clinical trials and formulation refinement are needed to enable TGP to meet the demand for the transition from preclinical evidence to clinical application, benefiting patients with liver cirrhosis.

## 1 Introduction

Liver cirrhosis represents the end-stage histopathological consequence of various chronic liver diseases, characterized by diffuse hepatic fibrosis, nodular architectural distortion, and progressive disruption of normal liver function. The condition imposes a substantial global health burden, accounting for nearly 1.5 million deaths annually worldwide. According to the Global Burden of Disease (GBD) 2021 database, the global incidence of liver cirrhosis reached 58.4 million cases in 2021, with total deaths attributable to cirrhosis escalating to 1,425,142. Notably, the overall incidence of liver cirrhosis has increased by 58.21% from 1990 to 2021, underscoring the growing public health challenge posed by this condition (1). Cirrhosis currently ranks as the 11th most common cause of death globally, and together with liver cancer, accounts for approximately 3.5% of all deaths worldwide. A recent systematic review and meta-analysis further estimated that the pooled global prevalence of advanced liver fibrosis and cirrhosis in the general population is 3.3% and 1.3%, respectively, with an upward trend observed after 2016. The etiological spectrum of cirrhosis has undergone a notable shift in recent decades. While viral hepatitis (particularly hepatitis B and C) and alcohol-related liver disease have historically been the dominant causes, non-alcoholic fatty liver disease (NAFLD)—now termed metabolic dysfunction-associated steatotic liver disease (MASLD)—has emerged as the leading global cause of incident cirrhosis (2). A meta-analysis reported that the pooled prevalence of cirrhosis among MASLD patients is 3.26% in general practice settings and as high as 14.51% among those in inpatient settings or referred for liver biopsy, with an all-cause mortality rate of 7.91 per 100 person-years. The growing contribution of metabolic etiologies, alongside persistent viral hepatitis and alcohol misuse in many regions, signals a critical shift in the global epidemiology of cirrhosis that demands renewed therapeutic strategies.

The gut-liver axis constitutes a bidirectional communication network that integrates microbial, metabolic, and immune signals between the gastrointestinal tract and the liver through the portal vein, biliary system, and systemic circulation. Emerging evidence has established that comprehensive disruption of the gut-liver axis is a pivotal driver in the pathogenesis and progression of advanced liver diseases, including cirrhosis (3, 4). In the cirrhotic state, intestinal dysbiosis—characterized by reduced commensal bacteria such as Bifidobacteria and Lactobacilli alongside expansion of potentially pathogenic Enterobacteriaceae and Veillonellaceae—impairs intestinal barrier integrity, leading to increased gut permeability (5–7). Consequently, microbial products, particularly lipopolysaccharide (LPS), translocate into the portal circulation and reach the liver, where they activate Kupffer cells via Toll-like receptor 4 (TLR4) signaling, triggering the release of pro-inflammatory cytokines such as tumor necrosis factor-alpha (TNF-α), interleukin-6 (IL-6), and interleukin-1 beta (IL-1β) (8). This inflammatory cascade drives hepatic stellate cell (HSC) activation, the principal effector of hepatic fibrogenesis, culminating in progressive collagen deposition and the establishment of cirrhosis. Simultaneously, the cirrhotic liver exhibits impaired bile acid synthesis and transport, which compromises the antimicrobial defense of the intestinal lumen and further exacerbates microbial dysbiosis, creating a self-amplifying vicious cycle. In parallel, accumulating evidence indicates that the NLRP3 inflammasome plays a critical role in this pathogenic circuit, as its aberrant activation in response to bacterial products and dysregulated bile acid signaling promotes sustained hepatic inflammation and fibrogenesis. Given this multifaceted pathophysiology, therapeutic approaches that can simultaneously restore intestinal barrier function, rebalance gut microbiota composition, normalize bile acid metabolism, and suppress hepatic inflammation through a unified mechanistic framework hold particular promise for the management of cirrhosis.

Total glucosides of paeony (TGP), the principal active extract derived from the dried root of Paeonia lactiflora Pall., has been used in traditional Chinese medicine for over 2,000 years. TGP primarily comprises paeoniflorin (accounting for more than 90% of total glucosides), along with albiflorin, oxypaeoniflorin, benzoylpaeoniflorin, and other related monoterpenoid glycosides. A growing body of pharmacological research has demonstrated that paeoniflorin exhibits a broad spectrum of hepatoprotective activities, including alleviation of cholestasis, attenuation of liver fibrosis, prevention of nonalcoholic fatty liver disease, and inhibition of hepatocellular carcinoma, mediated through multiple signaling pathways such as NF-κB, TGF-β1/Smads, PI3K/Akt/Nrf2, and JAK2/STAT6 (9). In particular, paeoniflorin has been shown to suppress hepatic stellate cell activation, reduce extracellular matrix deposition, and modulate macrophage polarization, thereby directly counteracting the fibrotic process within the liver. Ma et al.(2021) discovered through a streptozotocin-induced AD rat model that Albiflorin could reduce the Aβ deposition density in the hippocampus and regulate the Nrf-2/HO-1/HMGB1/NF-κB pathway related to inflammation and oxidative stress, indicating that Albiflorin has anti-inflammatory effects (10). Zhang et al. (2013)concluded through their research that paeniflorin and oxypaeniflora were able to attenuate advanced glycation end products-induced oxidative damage and inflammation in mesangial cells (11). Studies have shown that benzoylpaeoniflorin can inhibit the expression of key inflammatory mediators, making it a potential drug for treating sepsis and various inflammatory diseases (12). Similarly, albiflorin, oxypaeoniflorin, and benzoylpaeoniflorin have been found in related studies to inhibit the production of reactive oxygen species induced by lipopolysaccharide (LPS) in RAW264.7 cells, thereby exerting anti-inflammatory and antioxidant effects (13). Although TGP is a crude extract, the paeoniflorin it contains has shown protective effects on the liver in related pharmacological studies. Meanwhile, albiflorin, oxypaeoniflorin and benzoylpaeoniflorin may have certain protective effects on the liver through their pharmacological activities such as anti-inflammatory and antioxidant effects. These various components may interact synergistically, thereby helping to enhance the inhibitory effect on chronic liver cirrhosis. Beyond these direct hepatic actions, recent studies have unveiled that TGP also exerts profound regulatory effects on the intestinal microenvironment. TGP has been reported to remodel gut microbiota composition by enriching beneficial genera such as Lactobacillus, Akkermansia, Lachnospira, and Clostridium, while suppressing potentially harmful taxa, and to enhance intestinal barrier integrity through upregulation of tight junction proteins including Claudin-1, Occludin, and ZO-1 (14). Moreover, TGP may help maintain bile acid metabolic homeostasis and activate FXR signaling, which in turn may inhibit the NF-κB-mediated inflammatory cascade and suppress NLRP3 inflammasome activation—effects that collectively suggest a gut-centered mechanism of hepatic protection. Given that TGP targets multiple components of the gut-liver axis simultaneously—encompassing gut microbiota, intestinal barrier, bile acid metabolism, and hepatic inflammation—it represents a uniquely positioned therapeutic candidate for cirrhosis. Despite the accumulating evidence from preclinical studies, a systematic review that comprehensively integrates the current understanding of how TGP modulates the gut-liver axis to attenuate cirrhosis is still lacking.

This review aims to systematically summarize and critically evaluate the current state of knowledge regarding the therapeutic mechanisms of TGP in cirrhosis, with a particular focus on its multifaceted actions within the gut-liver axis. We propose that TGP may disrupts the self-perpetuating vicious cycle of cirrhosis through a hierarchical mechanism: restoration of intestinal barrier integrity, rebalancing of gut microbiota composition, normalization of bile acid metabolism, activation of FXR signaling, and dual suppression of NF-κB-driven inflammation and NLRP3 inflammasome activation. By integrating the available evidence from both experimental models and clinical investigations, this review seeks to provide a mechanistic framework for understanding TGP as a gut-liver axis modulator and to highlight emerging therapeutic opportunities as well as unresolved challenges in translating these findings into clinical practice for patients with cirrhosis.

## 2 Gut–liver axis dysregulation as a driver of cirrhosis

### 2.1 Overview of the gut–liver axis

The gut and liver engage in close bidirectional communication via the portal vein, the biliary system, and the systemic circulation, forming the so-called “gut–liver axis”. Under physiological conditions, the portal vein transports nutrients, microbial metabolites, and bacterial products from the intestine to the liver, while the liver, in turn, secretes bile acids and antimicrobial molecules into the intestinal lumen through the biliary system, thereby modulating the composition of the gut microbiota and maintaining intestinal ecological balance. This sophisticated interaction network not only ensures metabolic homeostasis but also plays a critical role in balancing systemic immune tolerance and defense against pathogens by regulating innate and adaptive immune responses. However, when disturbances occur in the gut microbiota, intestinal barrier function, or bile acid metabolism, the homeostasis of the gut–liver axis is disrupted, which can induce or exacerbate various liver diseases, particularly cirrhosis (15).

### 2.2 Gut microbiota dysbiosis in cirrhosis

In most healthy individuals, approximately 99% of the gut microbiota is composed of Firmicutes, Bacteroidetes, Proteobacteria, and Actinobacteria, with Firmicutes and Bacteroidetes together accounting for about 90%. When the diversity, composition, or function of the gut microbial community is disrupted, a state of dysbiosis develops, exerting negative effects on the host (16). In patients with cirrhosis, gut microbiota dysbiosis is characterized by a specific compositional shift: the abundance of Enterobacteriaceae, Streptococcaceae, Ruminococcaceae, and Proteobacteria increases, whereas that of Faecalibacterium, Clostridium, Coprococcus, and species of the order Clostridiales decreases (17, 18). Relevant studies have shown that compared with healthy individuals, patients with cirrhosis exhibit a marked reduction in Bacteroidetes and a significant increase in Proteobacteria and Fusobacteria (19). Enterobacteriaceae, as a hallmark of gut dysbiosis, are significantly enriched in the feces of cirrhotic patients and represent the primary group responsible for bacterial translocation, being extensively involved in systemic inflammatory responses (20).

This dysbiosis carries important pathological consequences. The reduction of beneficial bacteria, such as Firmicutes and Bacteroidetes, leads to the loss or weakening of essential functions that maintain intestinal barrier integrity and regulate the host immune system. Meanwhile, the increase in mucolytic and pathogenic bacteria directly damages the intestinal mucosal barrier, allowing more pathogens to invade the intestinal tissue (21). The reduced microbial diversity further weakens the gut’s resistance to colonization by pathogenic bacteria, establishing a vicious cycle.

### 2.3 Impaired intestinal barrier and bacterial translocation

The intestinal barrier is composed of the physical barrier (epithelial cells and their tight junctions), the chemical barrier (mucus layer, antimicrobial peptides), the immune barrier (gut-associated lymphoid tissue), and the microbial barrier (commensal microbiota). Among these components, tight junction proteins (including Claudin, Occludin, and ZO-1) form the most critical junctional complex at the apex of epithelial cells, precisely regulating transcellular transport and paracellular permeability.

In the cirrhotic state, gut microbiota dysbiosis leads to a reduction in beneficial bacteria and an increase in potentially harmful bacteria. This imbalance disrupts the tight junctions of the intestinal epithelium, thereby impairing intestinal barrier function and significantly increasing gut permeability. Viable bacteria and their products, especially endotoxins (lipopolysaccharide, LPS), translocate into the liver via the portal venous system. As pathogen-associated molecular patterns, these microbial products bind to Toll-like receptors (particularly TLR4), thereby activating downstream inflammatory signaling pathways (such as NF-κB) as well as the TGF-β signaling pathway. This activation induces Kupffer cells to release large amounts of pro-inflammatory cytokines, including tumor necrosis factor-α (TNF-α), interleukin-6 (IL-6), and interleukin-1β (IL-1β). These cytokines create an “inflammatory storm” within the liver, directly damaging hepatocytes and activating hepatic stellate cells (HSCs), which then transdifferentiate into myofibroblasts. Activated HSCs produce and deposit large quantities of collagen fibers while simultaneously suppressing collagen degradation, ultimately leading to liver fibrosis and cirrhosis. In addition, bacterial translocation contributes to the development of cirrhosis-related complications, such as spontaneous bacterial peritonitis.

### 2.4 Dysregulation of bile acid metabolism

Bile acids are key signaling molecules and metabolic hubs within the gut–liver axis. Their normal enterohepatic circulation begins in the liver, where cholesterol is used as a substrate to synthesize primary bile acids (cholic acid, CA, and chenodeoxycholic acid, CDCA). After conjugation with taurine or glycine, these primary bile acids are secreted into bile, stored in the gallbladder, and subsequently released into the intestine. In the gut, certain anaerobic bacteria (such as Firmicutes, Bacteroidetes, Eubacterium, and Clostridium) express bile salt hydrolases (BSHs) (22, 23), which deconjugate and dehydroxylate primary bile acids, converting them into secondary bile acids (deoxycholic acid, DCA, and lithocholic acid, LCA) (24, 25). Approximately 95% of bile acids (including both primary and secondary forms) are actively reabsorbed in the terminal ileum and returned to the liver via the portal vein, completing the cycle. Bile acids are not only essential for the absorption of dietary fats and fat-soluble vitamins but also act as endogenous ligands for the farnesoid X receptor (FXR) and the G protein-coupled receptor 5 (TGR5), thereby playing a broad role in regulating metabolism and maintaining internal homeostasis.

In cirrhosis, gut microbiota dysbiosis leads to a marked reduction in the conversion of primary bile acids to secondary bile acids. Specifically, alterations in the abundance of bile acid-metabolizing bacterial groups, such as Lachnospiraceae and Ruminococcaceae, reduce the diversity of the bile acid pool (26, 27). At the same time, the cirrhotic liver exhibits impaired capacity for bile acid synthesis and transport, further exacerbating bile acid metabolic disturbances. Because secondary bile acids are potent agonists of FXR, their decreased production results in inadequate activation of the FXR signaling pathway. FXR is essential for maintaining the homeostasis of the intestinal epithelial barrier and the gut-vascular barrier; impaired FXR signaling promotes bacterial translocation and aggravates liver injury (28, 29). Moreover, FXR suppresses *de novo* bile acid synthesis by inhibiting cholesterol 7α-hydroxylase (CYP7A1). When FXR activity is reduced, bile acid synthesis becomes uncontrolled, leading to the accumulation of toxic bile acids that further worsen liver damage.

### 2.5 NLRP3 inflammasome activation and hepatic fibrogenesis

The NLRP3 inflammasome is a multiprotein complex that assembles and becomes activated upon recognition of pathogen-associated molecular patterns and danger-associated molecular patterns, promoting caspase-1-mediated maturation and secretion of IL-1β and IL-18. In the pathogenesis of cirrhosis, the NLRP3 inflammasome plays a significant driving role through the gut–liver axis.

First, in the liver, toxic bile acids can directly activate the NLRP3 inflammasome pathway. Second, in the intestine, NLRP3 recognizes bacteria and their metabolites, initiating intestinal immune responses. When injurious stimuli persist, sustained inflammation leads to impairment of the intestinal mucosal barrier, which in turn triggers bacterial translocation, allowing large numbers of bacteria and their products to enter the portal circulation. Once bacteria translocate to the liver, the endotoxins they release further activate hepatic inflammatory responses. Chronic, low-grade inflammation promotes the transition of hepatic stellate cells from a quiescent to an activated state, leading to excessive production of extracellular matrix and deposition of collagen in the portal areas, thereby promoting fibrosis. As fibrosis progresses, the biliary tree within the liver becomes compressed, causing cholestasis. Prolonged exposure to toxic bile acids further aggravates hepatic inflammation and fibrosis, thus establishing a vicious cycle (30, 31).

Clinically, patients with cirrhosis exhibit a reduction in beneficial bacteria (Bacteroidetes, Firmicutes) and an increase in pathogenic bacteria (Streptococcus, Enterobacteriaceae). This microbial imbalance further amplifies the vicious cycle of intestinal barrier injury and hepatic inflammation. Animal studies have shown that NLRP3 gene knockout can partially reverse the metabolic disturbances associated with dysbiosis and alleviate liver fibrosis.

Notably, the farnesoid X receptor (FXR) exerts a critical negative regulatory role in this pathogenic network. On the one hand, FXR directly inhibits NLRP3 inflammasome activity by suppressing the phosphorylation of NLRP3 at serine 295, thereby blocking the development of hepatic inflammation and fibrosis. On the other hand, FXR reduces bile acid synthesis by inhibiting CYP7A1, thereby attenuating the activation of NLRP3 by bile acids at its source (32). Thus, the FXR-NLRP3 signaling axis represents a central node in the regulation of cirrhosis progression.

### 2.6 The vicious cycle of the gut–liver axis in cirrhosis

Taken together, the above discussion clearly delineates a self-amplifying vicious cycle of the gut–liver axis in cirrhosis. First, gut microbiota dysbiosis leads to a reduction in beneficial bacteria and an increase in pathogenic bacteria, which in turn disrupts the tight junctions of the intestinal epithelium, resulting in impaired intestinal barrier function and increased permeability. Consequently, bacteria and their endotoxins translocate into the liver via the portal vein. These microbial products activate the TLR4/NF-κB pathway in Kupffer cells, inducing the release of large amounts of pro-inflammatory cytokines (such as TNF-α, IL-6, and IL-1β). These cytokines further activate hepatic stellate cells and promote their transdifferentiation into myofibroblasts, leading to collagen deposition and liver fibrosis, which ultimately progresses to cirrhosis. In the cirrhotic state, the liver’s ability to deliver bile salts and antimicrobial molecules into the intestine through the biliary system is compromised, rendering it unable to effectively suppress excessive bacterial growth in the gut, thereby further exacerbating gut dysbiosis. Concurrently, dysregulation of bile acid metabolism results in suppressed FXR signaling, which removes the negative regulation of the NLRP3 inflammasome and perpetuates the amplification of inflammatory responses.

This vicious cycle reveals that once cirrhosis is established, it can continuously self-drive and worsen through the gut–liver axis. Therefore, any intervention capable of interrupting one or more steps in this cycle theoretically has the potential to slow or even reverse the progression of cirrhosis. This pathological foundation provides the essential rationale for exploring the therapeutic mechanisms of TGP in the subsequent sections (**Figure 1**).

**Figure 1:**
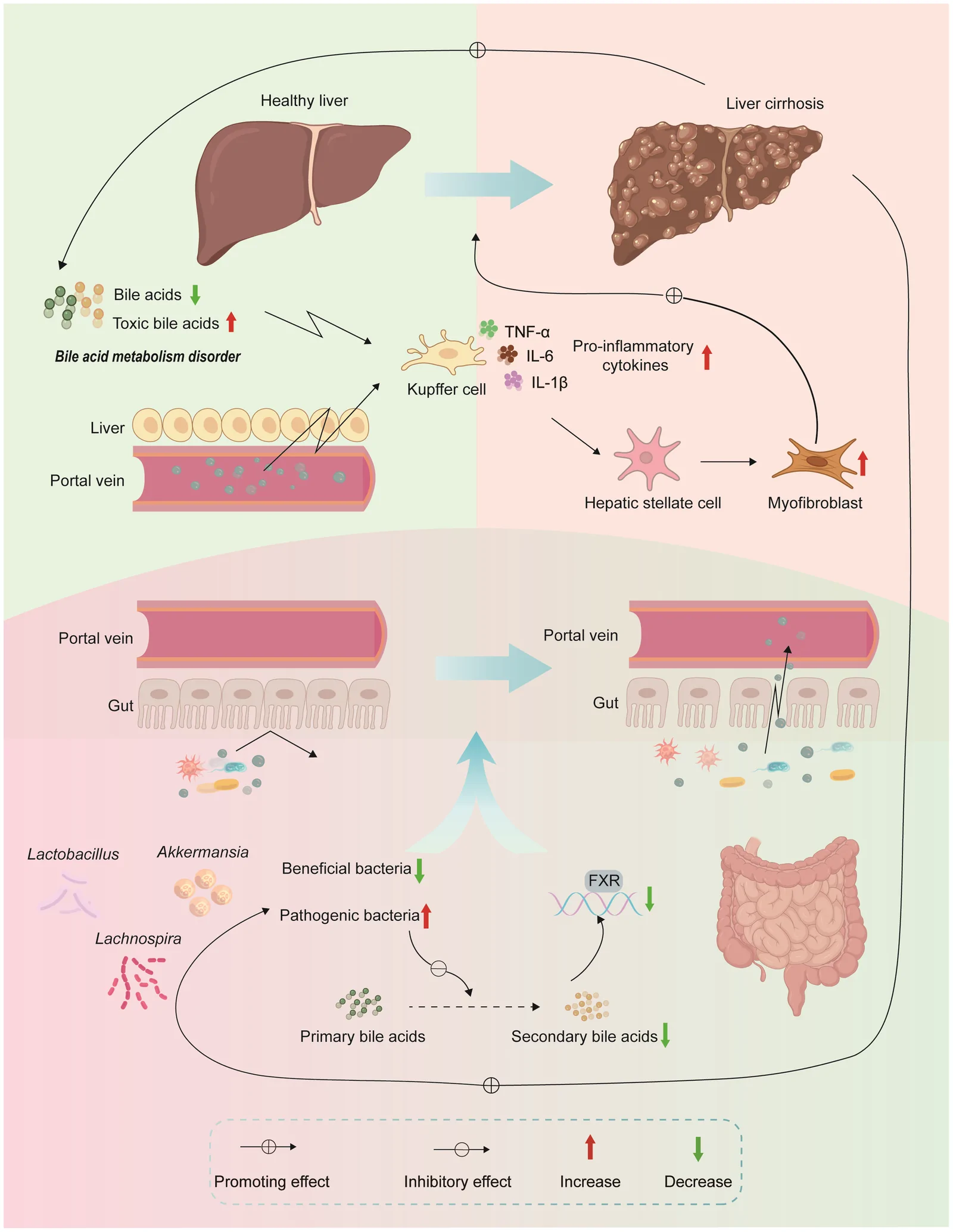
The vicious cycle of the gut–liver axis in cirrhosis.

## 3 TGP attenuates cirrhosis via gut–liver axis modulation

Building upon the delineation of the gut–liver axis vicious cycle in cirrhosis presented in the previous chapter, this chapter systematically discusses how TGP interrupts this vicious cycle through multi-target interventions, thereby counteracting the progression of cirrhosis. TGP is a multicomponent active extract derived from the dried root of Paeonia lactiflora Pall., mainly consisting of paeoniflorin, oxypaeoniflorin, albiflorin, hydroxypaeoniflorin, and benzoylpaeoniflorin, among which paeoniflorin accounts for more than 90% of the total glucosides and is the key component responsible for the pharmacological effects. TGP exerts a broad spectrum of biological activities, including anti-inflammatory, antioxidant, immunomodulatory, analgesic, cardiovascular protective, and gastrointestinal regulatory effects, through regulating the expression of multiple receptors and intracellular signaling pathways. In recent years, accumulating evidence has demonstrated that TGP exhibits significant hepatoprotective effects. Its interference with the gut–liver axis vicious cycle in cirrhosis can be systematically discussed from the following four aspects: reshaping of gut microbiota and restoration of intestinal barrier, normalization of bile acid metabolism and activation of FXR signaling, suppression of NF-κB-mediated inflammatory pathways, and blockade of NLRP3 inflammasome activation.

### 3.1 TGP reshapes gut microbiota composition

TGP intervention in the gut–liver axis vicious cycle in cirrhosis begins with the systemic regulation of gut microbiota composition and function. Numerous studies have confirmed that TGP significantly alters the diversity, abundance, and structural composition of the gut microbiota, characterized by enrichment of beneficial bacteria and suppression of potentially harmful bacteria.

Regarding beneficial bacteria, TGP markedly increases the abundance of several bacterial genera closely associated with host health, including Dubosiella, Lactobacillus, Akkermansia, Bacteroides, and Lachnospira. Among these, Lactobacillus, as a well-recognized producer of short-chain fatty acids (SCFAs), increases the intestinal content of butyrate and acetate when its abundance rises. SCFAs not only serve as a major energy source for intestinal epithelial cells but also enhance intestinal barrier function by up-regulating tight junction protein expression and exert anti-inflammatory effects by promoting the differentiation of regulatory T cells (33–37). Akkermansia, a bacterium capable of degrading mucin, has been shown to enhance tight junction protein expression and improve intestinal barrier integrity (38). Lachnospira has also been reported to maintain intestinal barrier integrity (39, 40). Regarding harmful bacteria, TGP effectively suppresses the proliferation of potentially pathogenic bacteria such as Ligilactobacillus and Mucispirillum. This “enriching beneficial, reducing harmful” optimization of the microbial community structure establishes a crucial microecological foundation for subsequent intestinal barrier repair and restoration of bile acid metabolic homeostasis.

It is worth noting that the gut microbiota-remodeling effect of TGP is not limited to cirrhosis models. TGP exhibits similar microbiota-regulating actions in various disease models. Wang et al. (2026) (41) demonstrated in a high-fat/sucrose diet-induced non-alcoholic fatty liver disease (NAFLD) mouse model that paeoniflorin treatment significantly reshaped gut microbiota composition, increased the abundance of the beneficial bacterium Akkermansia and microbe-derived SCFA levels, while suppressing the proliferation of pro-oxidant and pro-inflammatory pathogens such as Desulfovibrio and Helicobacter. Fecal microbiota transplantation experiments further confirmed that these antioxidant and metabolic improvements were mediated by the gut microbiota. Chen et al. (2025) (42) also showed in a mouse model of ulcerative colitis that TGP corrected gut microbial imbalance and, through a gut microbiota-dependent mechanism, inhibited the JAK2/STAT3 signaling pathway, thereby suppressing Th17 cell differentiation and alleviating colonic inflammation.

These studies indicate that the gut microbiota-reshaping effect of TGP is generalizable across different disease models, a property that confers a unique advantage to TGP in the intervention of chronic liver diseases such as cirrhosis. By restoring the diversity and functional integrity of the gut microbiota, TGP weakens the driving factors of the gut–liver axis vicious cycle at the source.

### 3.2 TGP restores intestinal barrier integrity via microbiota modulation

The integrity of intestinal barrier function depends on the normal structure and distribution of tight junction proteins between intestinal epithelial cells. Tight junction proteins (including Claudin, Occludin, and ZO-1) form the most critical junctional complex at the apex of epithelial cells, precisely regulating transcellular transport and paracellular permeability. In the cirrhotic state, gut microbiota dysbiosis leads to down-regulation of tight junction protein expression, impairment of barrier function, and increased permeability, thereby promoting bacterial and endotoxin translocation (43, 44). Through the microbiota-modulating effects described above, TGP significantly up-regulates the expression of tight junction proteins and repairs the damaged intestinal barrier.

The mechanism by which TGP enhances intestinal barrier integrity follows a three-tiered action pathway of “microbiota regulation—protein expression—barrier function”. At the microbiota level, Lactobacillus enriched by TGP produces SCFAs such as butyrate and acetate, which directly act on intestinal epithelial cells to promote the synthesis and assembly of tight junction proteins. Akkermansia has also been shown to enhance tight junction protein expression. At the protein expression level, studies have confirmed that TGP up-regulates the expression of key tight junction proteins including ZO-1, Occludin, and Claudin-3. In a study on TGP in neonatal rat necrotizing enterocolitis, TGP treatment significantly increased tight junction protein expression and improved intestinal barrier function. Meanwhile, TGP reduces the release of zonulin and pro-inflammatory cytokines such as IL-1β, TGF-β, and TNF-α, further decreasing intestinal permeability. In a mouse model of ulcerative colitis, TGP treatment enhanced intestinal barrier integrity by up-regulating colonic expression of ZO-1, Occludin, and claudin proteins. TGP also alleviated DSS-induced intestinal inflammation, repaired the intestinal mucosal barrier, and ameliorated gut microbial dysbiosis and metabolic imbalance.

By restoring the intestinal barrier, TGP effectively reduces the translocation of viable intestinal bacteria and their products, especially endotoxins, into the liver via the portal system, thereby intervening in the key link in the vicious cycle of cirrhosis: “dysbiosis → barrier impairment → bacterial translocation → hepatic inflammation”.

### 3.3 TGP normalizes bile acid metabolism and supports FXR signaling

Bile acids are key signaling molecules and metabolic hubs in the gut–liver axis. In cirrhosis, gut microbiota dysbiosis leads to a marked reduction in the conversion of primary bile acids to secondary bile acids, a decrease in bile acid pool diversity, and failure of normal FXR signaling pathway activation, which aggravates liver injury. TGP increases the abundance of beneficial bacteria such as Lactobacillus, thereby restoring the conversion capacity from primary bile acids (CA, CDCA) to secondary bile acids (DCA, LCA) and increasing colonic concentrations of DCA and LCA, thus restoring bile acid metabolic homeostasis.

More importantly, TGP can directly activate the FXR signaling pathway through multiple molecular mechanisms, thereby exerting hepatoprotective effects at various levels. First, TGP inhibits PKCα and MAPK pathway activation, leading to suppression of ERα activity. Since ERα can directly bind to the estrogen response element in the FXR promoter region and regulate its transcription, the inhibition of ERα activation relieves transcriptional suppression of FXR, thereby promoting FXR expression (45). Second, TGP increases SIRT1 expression; the deacetylation of FXR by SIRT1 promotes the formation of FXR/RXRα heterodimers, further enhancing the transcriptional activation capacity of FXR. Third, TGP directly protects against cholestatic liver injury through the SIRT1-FXR signaling pathway. Chen et al. (2021) demonstrated that paeoniflorin exerts protective effects against ANIT-induced cholestatic liver injury by activating the SIRT1-FXR signaling pathway (46). TGP also shows significant potential in regulating bile acid metabolism. A recent review systematically summarized the protective effects of TGP in liver and intestinal diseases, pointing out that TGP exhibits significant anti-inflammatory, antioxidant, anti-apoptotic, and bile acid metabolism-regulating effects through modulation of the SIRT1/FXR signaling pathway, and participates in gut–liver axis crosstalk by regulating FXR, bile acids, and the gut microbiota. Another review published in 2026 also emphasized the multiple actions of paeoniflorin in improving bile acid metabolism, inflammation, and oxidative stress (47).

Activated FXR exerts multifaceted protective functions in the treatment of cirrhosis. On the one hand, FXR directly inhibits the NF-κB inflammatory signaling pathway and indirectly suppresses NF-κB expression through the Nrf2/HO-1 pathway, thereby inhibiting intestinal and hepatic inflammatory responses (48–50). On the other hand, FXR reduces *de novo* bile acid synthesis by inhibiting cholesterol 7α-hydroxylase (CYP7A1), thereby attenuating toxic bile acid-induced liver damage at the source. Furthermore, FXR maintains the homeostasis of the intestinal epithelial barrier and the gut-vascular barrier, reducing bacterial translocation. Thus, by restoring bile acid metabolic homeostasis and activating FXR signaling, TGP achieves a “bidirectional regulation” within the gut–liver axis: upstream, it normalizes bile acid metabolism through microbiota modulation, and at the receptor level, it may enhance the activity of the FXR signaling pathway, thereby potentially counteracting the progression of cirrhosis.

### 3.4 TGP suppresses NF-κB and NLRP3-mediated inflammatory responses

Inflammation is one of the core drivers of the gut–liver axis vicious cycle. In cirrhosis, gut-derived endotoxins activate the TLR4/NF-κB pathway in Kupffer cells, leading to the release of large amounts of pro-inflammatory cytokines, which in turn activate hepatic stellate cells and drive liver fibrosis. TGP inhibits inflammatory responses at the two key nodes of NF-κB and NLRP3 through multiple mechanisms.

Regarding the NF-κB pathway, activated FXR directly suppresses the transcriptional activity of NF-κB. TGP also indirectly inhibits the NF-κB pathway by up-regulating SIRT1 expression. More importantly, TGP exerts direct anti-inflammatory effects by inhibiting the activation of the TLR4/NF-κB signaling pathway. Rong et al. (2025) found in a mouse model of sepsis-induced liver injury that paeoniflorin treatment dose-dependently reduced serum ALT and AST levels, ameliorated liver histopathological damage, decreased hepatocyte apoptosis, and significantly down-regulated the mRNA expression of pro-inflammatory cytokines (IL-6, TNF-α, IL-1β) in the liver. Further mechanistic studies revealed that TGP promoted the polarization of macrophages from the pro-inflammatory M1 phenotype to the anti-inflammatory M2 phenotype by inhibiting the activation of the TLR4/NF-κB signaling pathway. When the TLR4 agonist RS09 TFA was co-administered, the protective effect of paeoniflorin was completely abolished, confirming the critical role of the TLR4/NF-κB pathway in the anti-inflammatory action of paeoniflorin (51).

Regarding the NLRP3 inflammasome, TGP exhibits multi-level, multi-dimensional inhibitory effects. First, Feng Shu et al. constructed an FXR overexpression lentivirus and used a phosphorylated NLRP3 (serine 295 phosphorylated) antibody to detect the phosphorylation level of NLRP3 by protein immunoblotting. They found that FXR could inhibit the occurrence of liver fibrosis by suppressing the phosphorylation of NLRP3 (ser 295).Studies have shown that during cholestatic sepsis, bile acids, as DAMPs, can activate the NLRP3 inflammatory body signaling in macrophages. FXR plays a crucial role in controlling the NLRP3 inflammatory body in sepsis (52). Additionally, research has indicated that FXR can inhibit the activation of the NLRP3 inflammatory body induced by endoplasmic reticulum stress and alleviate liver damage (53). In patients with severe depression, the antidepressant lentinan A inhibits the activity of the NLRP3 inflammatory body by activating the bile acid receptor FXR, suppresses brain inflammation, and thereby exerts an antidepressant effect. In FXR knockout mice, the antidepressant effect of lentinan A disappears (54). These research results indicate that FXR has a negative regulatory effect on the activation of the NLRP3 inflammatory body. Therefore, we believe that TGP promotes the activation of the FXR signaling pathway. The activated FXR directly inhibits the activity of the NLRP3 inflammasome by suppressing the phosphorylation of NLRP3 at the serine 295 site, thereby blocking the liver inflammatory response and the formation of fibrosis. Second, by restoring bile acid metabolic homeostasis and activating FXR, TGP reduces the activation of NLRP3 by toxic bile acids. Third, TGP up-regulates SIRT1 expression, inhibits PKM2-mediated aerobic glycolysis, and reduces the activation level of NLRP3. Fourth, TGP blocks NLRP3 inflammasome activation at the source by inhibiting the accumulation of mitochondrial reactive oxygen species (mtROS). In a lipopolysaccharide plus D-galactosamine-induced acute liver injury model, a combined suspension of TGP and curcumin down-regulated the expression of TLR4, MyD88, IκBα, and NLRP3. The mechanism involved enhancement of antioxidant enzyme activities, inhibition of the TLR4/NF-κB/NLRP3 signaling pathway, and suppression of inflammasome assembly and release in liver tissue (55). Guo et al. (2025) further revealed that paeoniflorin could inhibit the NLRP3 inflammasome by targeting the STING-mediated pyroptosis pathway, thereby alleviating steatosis, oxidative stress, and inflammation in metabolic dysfunction-associated fatty liver disease (56).

Through these multifaceted mechanisms, TGP appears to concurrently suppress intestinal and hepatic inflammation by targeting two pivotal signaling nodes—NF-κB activation and NLRP3 inflammasome assembly—leading to significant attenuation of key pro-inflammatory cytokines, including IL-1β, IL-18, TNF-α, and IL-6. This coordinated inhibition suggests a mechanistic basis for interrupting the inflammatory crosstalk along the gut–liver axis.

### 3.5 Integrative mechanism: TGP disrupts the gut–liver axis vicious cycle

Taken together, the mechanisms by which TGP treats cirrhosis through the gut–liver axis can be summarized as a stepwise, synergistic cascade of actions.

At the upstream (intestinal) end of the gut–liver axis, TGP begins by systemically reshaping the gut microbiota, significantly increasing the abundance of beneficial bacteria such as Lactobacillus, Akkermansia, and Lachnospira while suppressing the proliferation of harmful bacteria. This optimization of the microbial community structure generates two parallel protective branches (57). At the receptor and signaling level (the central node), TGP further enhances FXR expression and activity by inhibiting the PKCα/MAPK pathway and up-regulating SIRT1. Activated FXR exerts three protective functions: directly inhibiting the NF-κB inflammatory pathway, indirectly inhibiting NF-κB through the Nrf2/HO-1 pathway, and reducing bile acid synthesis by inhibiting CYP7A1 while directly suppressing NLRP3 Ser295 phosphorylation.

At the inflammatory effector level (the downstream terminal), through the above mechanisms TGP achieves dual inhibition of NF-κB and the NLRP3 inflammasome. TGP can also block NLRP3 activation at the source by inhibiting aerobic glycolysis and mitochondrial reactive oxygen species, reducing the release of pro-inflammatory cytokines such as IL-1β, IL-18, TNF-α, and IL-6, thereby effectively alleviating intestinal and hepatic inflammation.

In summary, through the stepwise cascade of “repairing the intestinal barrier — restoring bile acid metabolism — activating FXR — dual inhibition of NF-κB and NLRP3” reveals the unique advantage of TGP as a multi-target natural product in the treatment of cirrhosis and provides a solid theoretical foundation for subsequent clinical translational research (**Figure 2**).

**Figure 2:**
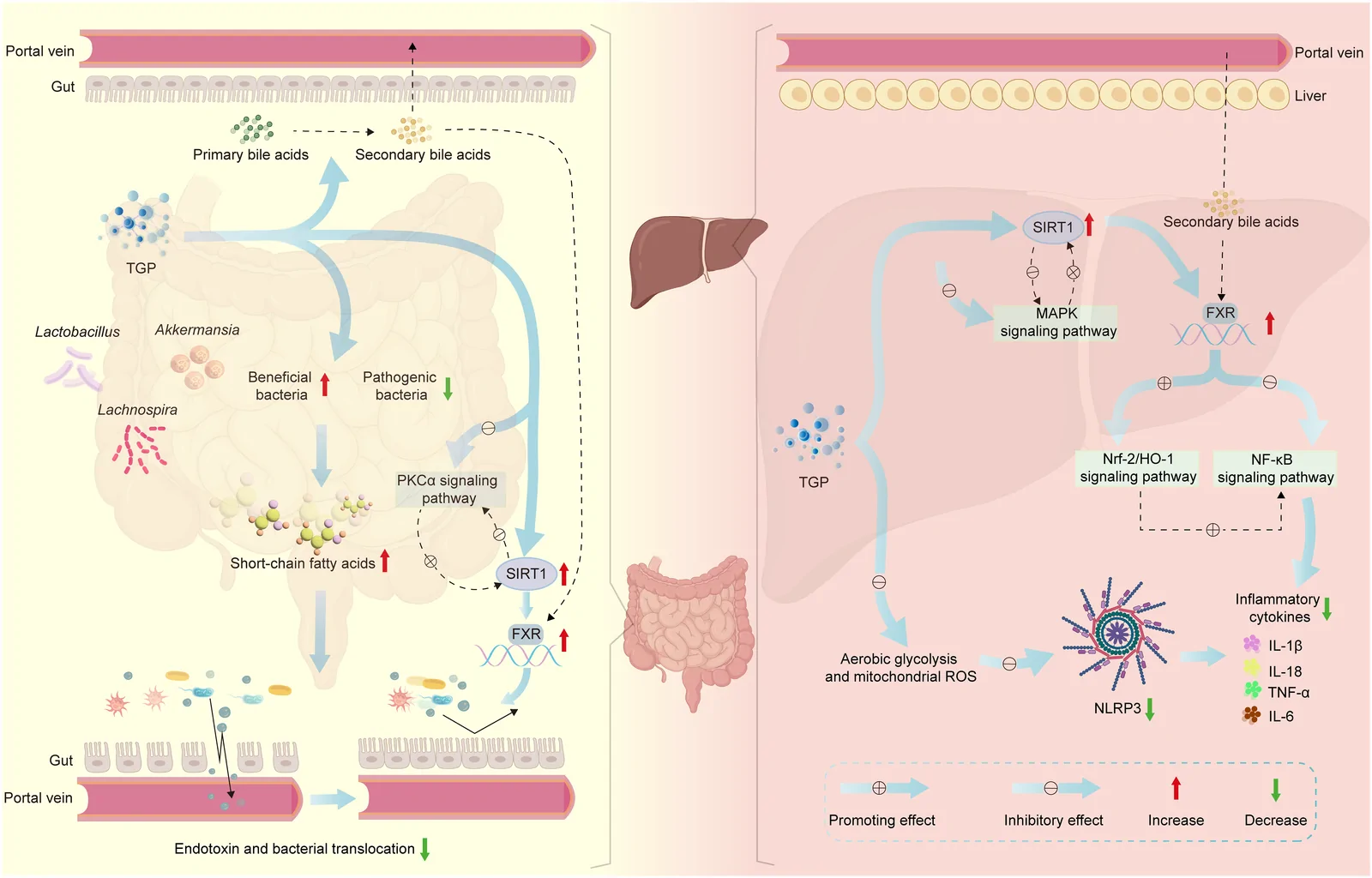
The mechanism of TGP in disrupting the vicious cycle of the intestinal-liver axis.

## 4 Discussion

This review systematically synthesizes current evidence that TGP ameliorates liver cirrhosis by modulating the gut–liver axis and proposes a stepwise mechanistic cascade. TGP reshapes the gut microbiota by enriching beneficial bacteria such as Lactobacillus, Akkermansia, and Lachnospira. On the one hand, these bacteria produce short-chain fatty acids that upregulate tight junction proteins, repair the intestinal barrier, and reduce portal translocation of endotoxins. On the other hand, TGP is associated with the conversion of primary to secondary bile acids, which may contribute to FXR signaling activation. Moreover, TGP further enhances FXR expression and activity by inhibiting the PKCα/MAPK pathway and upregulating SIRT1. Activated FXR not only directly suppresses the NF-κB inflammatory pathway but also inhibits NLRP3 Ser295 phosphorylation and reduces bile acid synthesis, achieving a dual blockade of inflammatory responses. This hierarchical cascade of “repairing the intestinal barrier — restoring bile acid metabolism — activating FXR — dual inhibition of NF-κB and NLRP3” may help interrupt the self-amplifying vicious cycle of cirrhosis, which runs from dysbiosis and barrier failure to bacterial translocation, hepatic inflammation, hepatic stellate cell activation, and ultimately fibrosis and cirrhosis.

Before discussing the multi-target advantages of TGP, a critical question is which of the aforementioned mechanisms reflect known FXR/NLRP3 regulatory networks and which are specific to TGP or paeony extracts. Meiya Li et al.(2019) conducted experiments by measuring the methylation level of the ERα promoter, assessing the expression of DNMT, verifying through the use of methylation inhibitors, and observing the renal injury and inflammatory indicators in SLE mice. They found that TGP can inhibit the autoimmune response in SLE by down-regulating the expression of ERα (58). TGP can inhibit the activity of ERα. And relevant studies have shown that inhibiting the activation of ERα can relieve the transcriptional inhibition on FXR, thereby promoting the expression of FXR. TGP promotes the expression of FXR by inhibiting the activation of ERα. This is the first report by TGP on the activation mechanism of FXR. Activated FXR directly inhibits the activity of NLRP3 inflammation complex by suppressing the phosphorylation of NLRP3 at the serine 295 site, which is a verification of the known pathway. Currently, there is no evidence of TGP directly regulating the phosphorylation of NLRP3.

Mechanistically, the multi-target profile of TGP aligns well with the complex pathophysiology of cirrhosis. Unlike single-target agents (e.g., pure portal pressure reducers or pure anti-inflammatory drugs), TGP simultaneously targets the upstream (microbiota and barrier), midstream (bile acids and FXR), and downstream (inflammatory pathways) components of the gut–liver axis. Notably, the gut microbiota-modulating action of TGP is characterized by a genuine “positive remodeling”—it not only suppresses harmful bacteria but, more importantly, enriches beneficial taxa closely linked to host health. This action resembles that of prebiotics, but with a broader regulatory spectrum and additional direct effects on host signaling (e.g., FXR activation). Compared with obeticholic acid, a direct FXR agonist that often causes dose-dependent pruritus and dyslipidemia (59), TGP may promote FXR through both microbiota-dependent and microbiota-independent pathways, potentially achieving more physiological regulation with fewer adverse effects. Furthermore, TGP already has a favorable safety profile from clinical use in autoimmune diseases such as rheumatoid arthritis, providing a basis for long-term application in a chronic condition like cirrhosis (60).

Nevertheless, the available evidence has important limitations. Firstly, the majority of the mechanism studies we have conducted are based on single cell lines or acute injury models (e.g., CCl_4_-induced fibrosis, bile duct ligation, methotrexate or LPS/D-GalN-induced acute liver injury). Although these models can reproduce some characteristics of liver cirrhosis, they do not fully represent the metabolic, immune and microbiological complexity of human liver cirrhosis. These models may not accurately reflect the chronic progressive pathological process of liver cirrhosis. For instance, the gut microbiota composition and bile acid kinetics differ substantially between mice and humans. Therefore, the actual efficacy of TGP in liver cirrhosis patients still needs to be verified. We will next conduct further research on the entire course of liver cirrhosis. Second, pharmacokinetic data are severely lacking. TGP has low oral bioavailability (approximately 3-4%) due to extensive gut microbial metabolism and efflux. Although relevant studies have found that other components in TPG have a positive interaction with the absorption and retention of paeonol (61). But the pathological state of cirrhosis—characterized by portal hypertension, intestinal congestion, and compromised liver function—may profoundly alter the absorption, distribution, metabolism, and excretion of TGP. Sun Xiaoyu et al. investigated the impact of portal hypertension on intestinal drug absorption and found that in the portal hypertension rat model, the intestinal absorption function was impaired. The mechanism was related to the upregulation of intestinal efflux transporters P-gp, MRP2, and metabolic enzyme CYP3A4, which increased the intestinal first-pass effect of drugs and thereby limited absorption (62). However, there are currently no pharmacokinetic studies of TGP in patients with liver cirrhosis. Third, the differential contributions of individual TGP components (paeoniflorin, albiflorin, oxypaeoniflorin, etc.) are unclear. Most studies focus on paeoniflorin, whereas whether the other components exert synergistic or antagonistic effects remains unknown. Fourth, long-term safety data are insufficient. Patients with cirrhosis often require prolonged or lifelong medication, and the effects of chronic TGP use on bile acid homeostasis, hepatic transporter function, and the gut microbial ecosystem have not been evaluated.

At present, there is no clinical research registration for TGP’s indications for liver cirrhosis. The existing registered trials mainly focus on indications such as autoimmune hepatitis, psoriasis, and vitiligo. Possible reasons include: Firstly, clinical trials for liver cirrhosis face difficulties in endpoint selection, large population heterogeneity, diverse causes, and challenges in ethical intervention and control design. These factors significantly increase the threshold and cost of registration trials. Secondly, the evidence related to TGP for liver diseases currently mainly comes from animal models and cell mechanism studies, as well as small-scale, single-center exploratory clinical observations. There are multiple gaps between “academic exploration” and “registration-based clinical development”, including evidence volume, research standardization, and the willingness of the sponsor to invest. Currently, the preclinical evidence chain of TGP for liver diseases has been initially established. Relevant reviews have also systematically summarized the pharmacodynamic evidence of TGP in acute liver injury, non-alcoholic fatty liver disease, and chronic liver fibrosis, providing directions for preclinical research on liver cirrhosis. TGP has been clinically discovered for treating liver cirrhosis, and there is a very broad space for its clinical translation advancement.

However, there are also some challenges in the clinical translation process, such as: Firstly, there is currently a lack of systematic data on combined pharmacokinetics and pharmacodynamics research. Secondly, the etiological heterogeneity of liver cirrhosis (viral, alcoholic, metabolic, autoimmune) is strong, and the fibrosis driving mechanisms under different etiologies are different. The hard endpoints of liver cirrhosis clinical trials (such as decompensation events, liver transplantation, all-cause death) require 3–5 years of follow-up and a large sample size to achieve sufficient statistical power, while histological endpoints (such as fibrosis reversal) rely on liver biopsies, which have high operational risks and low patient acceptance, directly raising the investment threshold and uncertainty of registration trials.

To advance clinical translation, future research should prioritize several directions. First, well-designed clinical trials are needed. Exploratory studies in patients with compensated cirrhosis should assess endpoints such as intestinal permeability (lactulose/mannitol ratio), serum endotoxin levels, inflammatory markers, and liver function tests. After obtaining preliminary evidence, large-scale randomized controlled trials using fibrosis reversal (transient elastography or biopsy), decompensated events (ascites, variceal bleeding, hepatic encephalopathy), and survival as endpoints should be conducted. Second, germ-free animal models and fecal microbiota transplantation experiments should be used to dissect the microbiota-dependent versus microbiota-independent effects of TGP, and multi-omics approaches (metagenomics, metabolomics) should identify key active metabolites. Third, targeted delivery formulations (e.g., enteric-coated or nano-encapsulated TGP) should be developed to increase local intestinal concentration and bioavailability of TGP. Fourth, synergistic combinations with existing anti-fibrotic drugs (e.g., obeticholic acid), probiotics, or intestinal absorbents (e.g., rifaximin) should be explored. Finally, precision medicine strategies based on baseline gut microbiota composition, bile acid profile, and genetic background could identify patient subpopulations most likely to benefit from TGP therapy.

In summary, the integrated mechanistic framework proposed in this review provides a theoretical basis for the use of TGP in cirrhosis via the gut–liver axis. Although current evidence is predominantly preclinical, TGP, as a multi-target natural product, may offer unique potential to help interrupt the vicious cycle of cirrhosis. Addressing the limitations outlined above and translating basic findings into clinical validation represent key priorities for future research.

## 5 Conclusions

Liver cirrhosis is the end-stage consequence of various chronic liver diseases, and its development is closely associated with dysregulation of the gut–liver axis. Gut microbiota dysbiosis, impaired intestinal barrier, bacterial endotoxin translocation, dysregulated bile acid metabolism, suppression of FXR signaling, and activation of the NLRP3 inflammasome intertwine to form a self-amplifying vicious cycle that drives hepatic inflammation, fibrosis, and ultimately cirrhosis. TGP has a complex composition, and existing research mainly focuses on paeoniflorin, but albiflorin, oxypaeoniflorin and benzoylpaeoniflorin may have certain protective effects on the liver through their pharmacological activities such as anti-inflammatory and antioxidant effects. These various components may interact synergistically, thereby helping to enhance the inhibitory effect on chronic liver cirrhosis. As a multi-component natural product, total glucosides of paeony may be capable of interrupting this vicious cycle through systematic multi-target intervention. Specifically, TGP reshapes the gut microbiota composition by increasing the abundance of beneficial bacteria such as Lactobacillus and Akkermansia. On the one hand, these beneficial bacteria produce short-chain fatty acids that up-regulate the expression of tight junction proteins, repair the intestinal barrier, and reduce bacterial translocation. On the other hand, TGP may help maintain bile acid metabolic homeostasis and promotes the FXR signaling pathway. Meanwhile, TGP may further enhance FXR expression and activity by inhibiting the PKCα/MAPK pathway and up-regulating SIRT1. Activated FXR not only suppresses the NF-κB inflammatory pathway but also directly inhibits NLRP3 Ser295 phosphorylation and reduces bile acid synthesis, thereby dually blocking inflammatory responses. Ultimately, through the stepwise cascade of “repairing the intestinal barrier — restoring bile acid metabolism — activating FXR — dual inhibition of NF-κB and NLRP3”cascade reaction mechanism, the progression of liver cirrhosis is delayed. However, the current mechanism model is mainly based on the synthesis of various studies rather than conducting causal verification within the same experimental system. This model remains a “hypothesis-driven integrated framework”. In the future, we need to further verify it through designing experiments such as FXR gene knockout, removal of the microbiome, and time-series analysis of metabolomics, etc. Although current research is mainly limited to the preclinical stage, TGP shows unique advantages and potential in the treatment of cirrhosis. Future research should focus on clinical validation, mechanistic deepening, and formulation optimization, enabling TGP to meet the demand for the transition from preclinical evidence to clinical application and ultimately benefiting patients with liver cirrhosis.
